# Minimally Invasive Revision of Luque Plate Instrumentation: A Case Report

**DOI:** 10.7759/cureus.53120

**Published:** 2024-01-28

**Authors:** Peter B Derman, Mary P Rogers-LaVanne, Alexander M Satin

**Affiliations:** 1 Department of Spine Surgery, Texas Back Institute, Plano, USA; 2 Department of Research, Texas Back Institute, Plano, USA

**Keywords:** spinal stenosis, spinal fusion, reoperation, pedicle screws, lumbar vertebrae, bone plates

## Abstract

Extension of existing spinal fusions may necessitate the removal of or linkage to prior constructs. Knowledge of previously placed instrumentation is critical to success in these revision scenarios. The Luque spinal instrumentation system, developed in the late 1980s, is a legacy pedicle screw and plate system that may be encountered during revision operations today.

A 67-year-old male with a remote history of L4-S1 fusion with Luque instrumentation presented with bilateral lower extremity neurogenic claudication due to adjacent segment disease at L3-4. Decompression and extension of fusion to the L3-4 level were performed using minimally invasive techniques. Of note, posterior instrumentation was extended by removing prior L4 pedicle screws with a 7 mm female hexagonal driver through tubular retractors, leaving the Luque plates in place, placing modern pedicle screws at L4 (through the plates) and L3, and linking these with standard rods. The surgery and post-operative course were uncomplicated, and the patient experienced complete resolution of his pre-operative claudication symptoms. Extension of prior Luque plate instrumented fusion can be accomplished minimally invasively without removing the plates themselves, resulting in greater operative efficiency and less surgical morbidity.

## Introduction

The volume of lumbar fusion operations has increased in recent decades [1-2]. A subset of these patients eventually require surgery for adjacent segment disease [3]. A variety of techniques exist for extending fusion to additional levels; these may involve the removal of or linking to prior posterior instrumentation. Pre-operative planning with a thorough knowledge of the previously placed implants is critical for success in these revision cases.

The Luque spinal instrumentation system, introduced in 1988 and produced by Danek, was a semirigid plate-and-screw device [4]. It utilized pedicle screws inserted through an open-loop plate with plate rings designed to prevent plate spreading [5-6]. The screws had spherical undersides that were seated in corresponding plate concavities, but the two were not rigidly fixed [5]. Screws were available in 5.5 mm and 6.5 mm diameters, and contourable plates came in 3-15 cm lengths in 1 cm increments [5]. This system was eventually retired and is no longer commercially available.

In this case report, a minimally invasive method for extension of fusion in the setting of Luque instrumentation is presented with the goal of providing pertinent information on the management of this legacy spinal system in the setting of revision surgery.

The case report was determined to be exempt from institutional review board review. The patient was informed and provided consent that data and images concerning the case be submitted for publication.

## Case presentation

Presentation

A 67-year-old male presented to the office with bilateral lower extremity neurogenic claudication. He had a history of two prior uncomplicated lumbar posterior decompressions and fusions, one in 1991 and a second in 1999. He did well until two years prior to presentation when his ambulatory capacity began progressively declining. Symptoms persisted despite conservative care.

**Figure 1:**
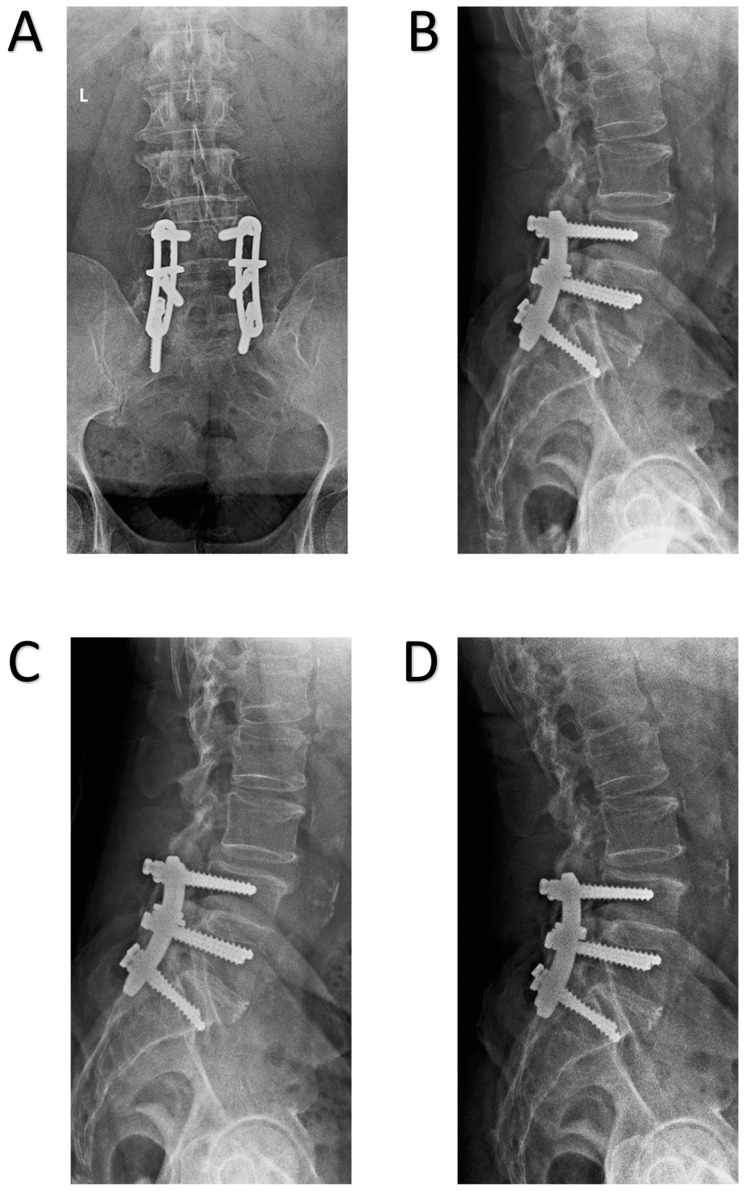
Upright AP (A), lateral (B), lateral flexion (C), and lateral extension (D) radiographs of the lumbar spine on presentation revealed intact Luque implants at L4-S1 with mobile Grade 1 degenerative anterolisthesis at L3-4 AP: anteroposterior

**Figure 2:**
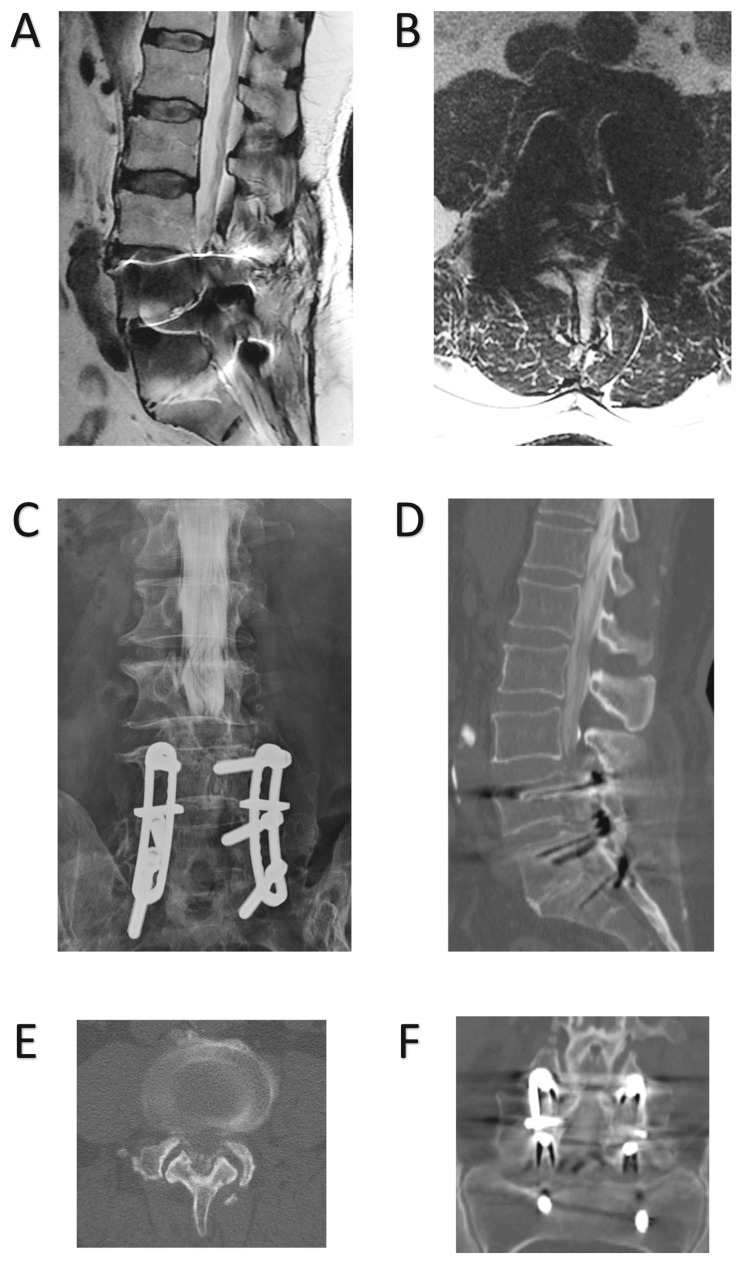
Advanced imaging findings on presentation (A-B) Mid-sagittal T2-weighted MRI (A) as well as a corresponding axial cut through the L3-4 disc space (B) demonstrated adjacent segment degeneration with L3-4 canal stenosis. (C-E) Myelogram (C), mid-sagittal (D), and L3-4 axial (E) cuts from a subsequent CT with similar findings. (F) CT myelogram also confirmed successful L4-S1 posterolateral fusion, which can be appreciated in this coronal image MRI: magnetic resonance imaging, CT: computed tomography

On physical examination, he was neurologically intact with a healed midline posterior incision over his lumbosacral spine. Radiographs revealed a previous L4-S1 posterolateral fusion with Luque instrumentation and mobile Grade 1 degenerative L3-4 anterolisthesis (Figure 1). A lumbar spine MRI as well as a myelogram with subsequent CT were performed (Figure 2). These confirmed successful L4-S1 fusions, revealed L3-4 spondylosis and associated severe canal stenosis, and demonstrated no other notable sites of stenosis throughout. The risks and benefits of surgical intervention were fully discussed with the patient, and the patient consented to surgical treatment. The decision was made to proceed with L3-4 decompression and fusion.

Operative details

Surgery began with L3-4 lateral lumbar interbody fusion, which proceeded uneventfully via a standard left-sided approach in the lateral decubitus position. An expandable cage packed with recombinant human bone morphogenetic protein-2 was deployed. This incision was closed, and the patient was repositioned prone on a Jackson frame.

**Figure 3:**
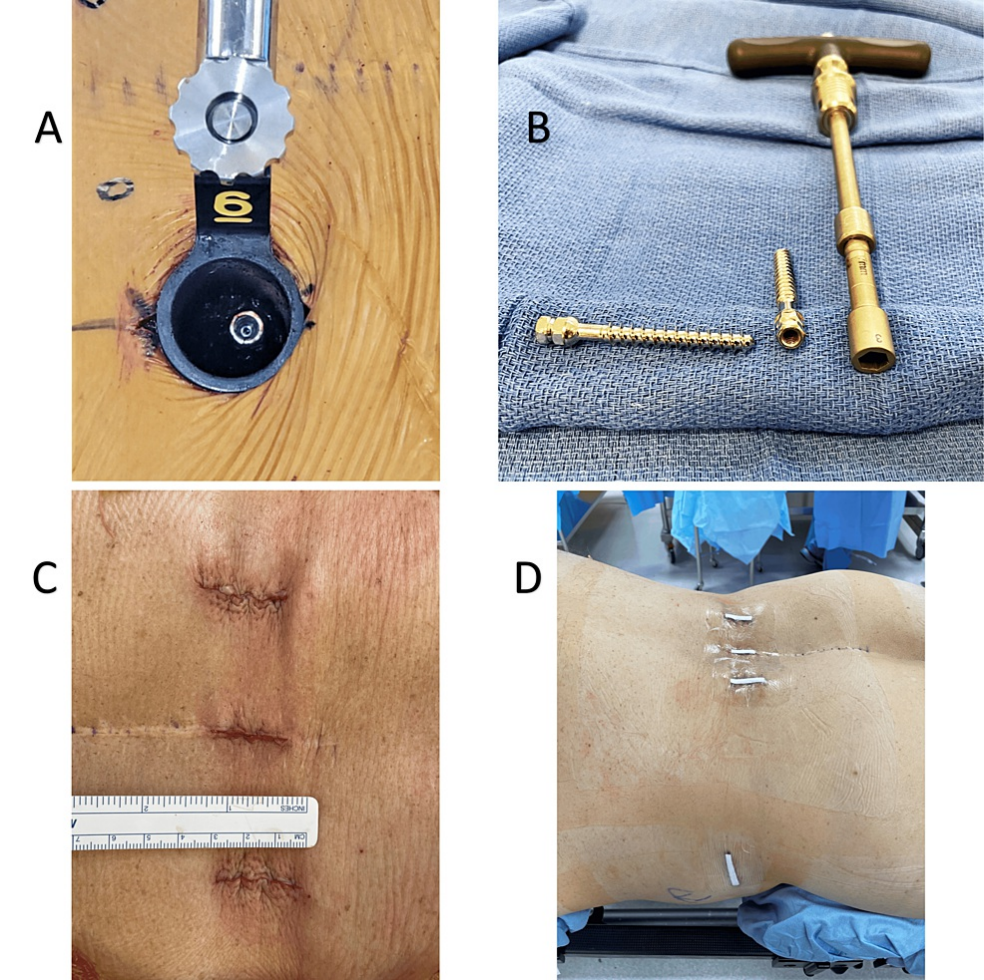
Intra-operative images (A) View of the left L4 Luque pedicle screw head seen through the tubular retractor. (B) Explanted pedicle screws with corresponding removal driver. (C) Closed posterior incisions; the Wiltse incisions were used for L4 Luque pedicle screw explantation and extension of instrumented posterolateral fusion, and the midline incision was used for decompression. (D) Final dressings

Fluoroscopy was used to guide the placement of 3 cm bilateral Wiltse incisions for access to the L3 pedicles and the rostral aspect of the prior construct. Starting on the left, sequential dilators were advanced onto the previously placed L4 pedicle screw, followed by a tubular retractor, which was then secured to the operating table. The left L4 pedicle screw head was exposed (Figure 3A). A 7 mm female hexagonal driver from a universal hardware removal set was found to fit snugly over the screw head and used to remove this pedicle screw (Figure 3B).

The original plan was to then cut the plate with a carbide metal-cutting drill bit above the L5 pedicle screws through the tubular retractor, remove the rostral plate fragment, and then perform segmental L3-4 instrumented posterolateral fusion. However, the Luque plate was found to be encased within a considerable posterolateral fusion mass. There was concern that the amount of bone removal required to access and cut the plate might compromise the integrity of the L4-5 fusion, necessitating more extensive surgery than segmental L3-4 posterior instrumentation. This plan was therefore aborted. Consideration was given to the extension of the Wiltse incisions for additional plate exposure and possible complete posterior implant removal, but a less invasive technique was preferred. Instead, a flexible guidewire was placed down the prior left L4 pedicle tract, the tubular retractor was removed, and the identical process was performed on the right side.

The bilateral L3 pedicles were then cannulated via the Wiltse incisions using a Jamshidi and fluoroscopy. Flexible guidewires were placed, and attention was turned to the decompression. A 2 cm portion of the prior midline incision was opened overlying the L3-4 level. A right L3-4 unilateral hemilaminotomy for bilateral decompression was performed through a tubular retractor using a standard technique [7-9]. The retractor was then redirected more laterally over the right L3-4 facet joint, which was thoroughly decorticated using a burr. Local bone was packed into the joint to promote posterolateral fusion. The retractor was removed.

**Figure 4:**
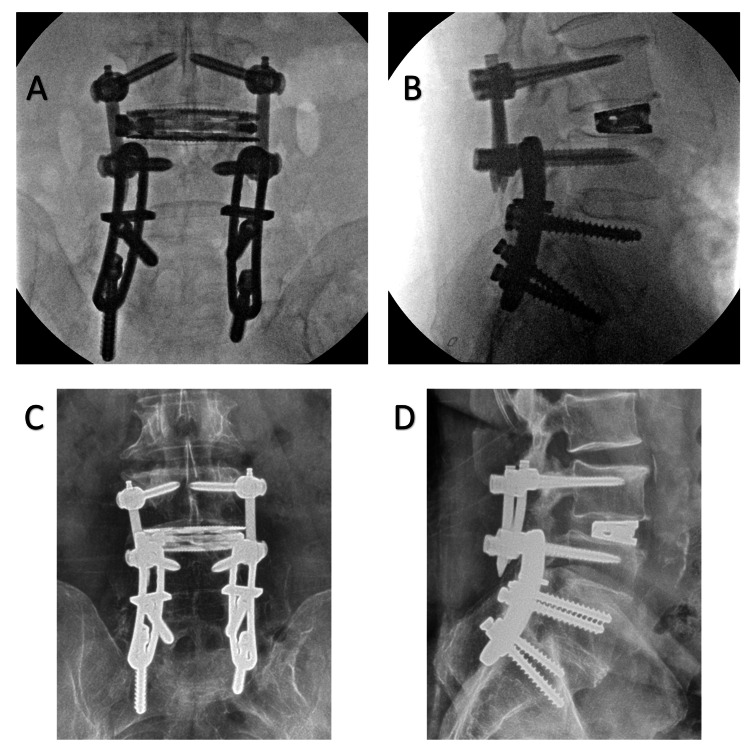
(A-B) Intra-operative AP (A) and lateral (B) fluoroscopic images demonstrating the final construct. (C-D) Upright AP (C) and lateral (D) radiographs at one-year follow-up reveal intact implants without suggestion of loosening or failure AP: anteroposterior

Cannulated, minimally invasive pedicle screws (Creo MIS, Globus Medical, Inc., Audubon, PA) were then placed over the guidewires. At the L4 level, 6.5-mm-diameter screws were found to pass through the prior Luque plates and provide excellent purchase in the underlying bone. The L3 screws were intentionally left slightly proud so as to align with the L4 screws, which could not be advanced further given the presence of the plates. Screw lengths were upsized slightly (50 mm length at both L3 and L4) to accommodate this. Rods were passed, final tightening performed, screw tabs were removed, and wounds were irrigated and closed (Figure 3C-3D). Final fluoroscopy shots demonstrated the completed construct (Figure 4A-4B). The estimated blood loss was 50 mL.

Post-operative course

The post-operative course was uncomplicated. The patient was discharged home after one night in the hospital (<24-hour length of stay). He experienced complete resolution of his neurogenic claudication and was off all pain medications within three weeks. He continued to do well at the most recent follow-up (one year out from surgery) with neither significant pain nor functional limitation. Post-operative radiographs revealed maintained implant placement without evidence of loosening, migration, subsidence, or failure (Figure 4C-4D).

## Discussion

Like every type of spinal instrumentation, the Luque plate-and-screw system has unique features that impact surgical strategies during revision surgery. This technical case report describes the details of removal and a minimally invasive strategy for extension-information that, to the authors’ knowledge, is not otherwise available in the modern literature. Hadgaonkar et al. published a report on the revision of two cases with Steffee plate instrumentation, which is a similar but not identical system [10]. Their technique involved an open approach to plate removal.

One potential downside of our technique is that the newly placed implants may sit slightly proud compared to a case in which the Luque plate was removed. This could potentially result in hardware prominence and discomfort, especially in thin patients. The patient described here has not experienced such issues, but modern implants could theoretically be removed in a symptomatic patient once osseous fusion has occurred. Of note, this technique represents off-label use of the described implants. While theoretical concerns regarding galvanic corrosion in constructs combining implants composed of different metal alloys have been raised, such issues have not been observed in the literature [11-12].

This case report is not without limitations. It demonstrates the feasibility of this surgical strategy, but only the outcome of a single case. Furthermore, modern 6.5 mm screws were inserted through the Luque plates, as these were felt to be appropriately sized for the patient’s anatomy. No attempt was made to place other screw sizes, so it remains unknown to the authors whether larger-diameter screws would pass through the plates.

## Conclusions

Luque plate-and-screw instrumentation is no longer commercially available but may be encountered by spine surgeons in a revision scenario. The described case report provides critical information for the management of this legacy spinal system in the setting of revision surgery. Using minimally invasive techniques, prior pedicle screws can be removed with a 7 mm female hexagonal driver, the plates left in place, and instrumentation extended by inserting modern screws through the existing plates.

## References

[1] Singh R, Moore ML, Hallak H (2022). Recent trends in medicare utilization and reimbursement for lumbar fusion procedures: 2000-2019. World Neurosurg.

[2] Lambrechts MJ, Siegel N, Heard JC (2022). Trends in single-level lumbar fusions over the past decade using a national database. World Neurosurg.

[3] Joelson A, Sigmundsson FG (2022). Additional operation rates after surgery for degenerative spine diseases: minimum 10 years follow-up of 4705 patients in the national Swedish spine register. BMJ Open.

[4] Luque ER, Rapp GF (1988). A new semirigid method for interpedicular fixation of the spine. Orthopedics.

[5] Trammell TR, Rapp G, Maxwell KM, Miller JK, Reed DB (1991). Luque interpeduncular segmental fixation of the lumbosacral spine. Orthop Rev.

[6] Zdeblick TA (1993). A prospective, randomized study of lumbar fusion. Preliminary results. Spine (Phila Pa 1976).

[7] Spetzger U, Bertalanffy H, Naujokat C, von Keyserlingk DG, Gilsbach JM (1997). Unilateral laminotomy for bilateral decompression of lumbar spinal stenosis. Part I: anatomical and surgical considerations. Acta Neurochir (Wien).

[8] Spetzger U, Bertalanffy H, Reinges MH, Gilsbach JM (1997). Unilateral laminotomy for bilateral decompression of lumbar spinal stenosis. Part II: clinical experiences. Acta Neurochir (Wien).

[9] Balsano M, Härtl R, Hussain I (2023). Microscopic Tubular Unilateral Laminotomy for Bilateral Decompression (MT-ULBD). AO Surgery Reference.

[10] Hadgaonkar S, Vincent V, Rathi P, Sancheti P, Shyam A (2021). Revision of Steffee plate instrumentation - challenges and technical tips. Interdiscip Neurosurg.

[11] Denduluri SK, Koltsov JC, Ziino C (2021). Rod-screw constructs composed of dissimilar metals do not affect complication rates in posterior fusion surgery performed for adult spinal deformity. Clin Spine Surg.

[12] Serhan H, Slivka M, Albert T, Kwak SD (2004). Is galvanic corrosion between titanium alloy and stainless steel spinal implants a clinical concern?. Spine J.

